# Draft Genome Sequence of Urease-Producing *Pseudorhodobacter* sp. Strain E13, Isolated from the Yellow Sea in Gunsan, South Korea

**DOI:** 10.1128/MRA.00189-19

**Published:** 2019-06-06

**Authors:** Si Chul Kim, Hyo Jung Lee

**Affiliations:** a Department of Biology, Kunsan National University, Gunsan, Republic of Korea; Indiana University, Bloomington

## Abstract

Here, we report the draft genome sequence of Pseudorhodobacter sp. strain E13, a Gram-negative, aerobic, nonflagellated, and rod-shaped bacterium which was isolated from the Yellow Sea in South Korea. The assembled genome sequence is 3,878,578 bp long with 3,646 protein-coding sequences in 159 contigs.

## ANNOUNCEMENT

The genus *Pseudorhodobacter*, member of the Rhodobacteraceae of the Alphaproteobacteria, was reclassified from Agrobacterium (1) by Uchino et al. (2). The genus *Pseudorhodobacter* differs from the genus Rhodobacter in that it does not have complete genes for photosynthesis (3). Currently, the genus *Pseudorhodobacter* contains nine species isolated from various environments, such as seawater (4), freshwater (5), and soil (6). However, it is not known what function they play in nature.

*Pseudorhodobacter* sp. strain E13 was isolated from the Yellow Sea, located west of Gunsan in South Korea (35°53′21″N, 126°30′40″E) on 4 January 2018 using a previously described procedure with some modifications (7). Briefly, a seawater sample was serially diluted with phosphate-buffered saline (PBS) buffer, spread on marine agar (Difco 2216), and incubated at 25°C for 5 days under aerobic conditions. A pure colony of strain E13 was obtained and inoculated into marine broth (MB). Genomic DNA was extracted from a 1-ml culture, grown in MB at 25°C for 48 h, using the Promega Wizard DNA purification kit (Qiagen) according to the manufacturer’s instructions. The DNA libraries prepared with the TruSeq DNA PCR-free kit according to the TruSeq DNA PCR-free sample preparation guide. The genome of *Pseudorhodobacter* sp. E13 was sequenced with an Illumina HiSeq X Ten sequencer with 150-bp paired-end reads at Macrogen (South Korea). The resulting 2,535,453,986 bp with 16,791,086 total reads (650-fold coverage) were trimmed using Sickle in paired-end mode (8) and assembled using SOAPdenovo (v 2.01) with a k-mer (9). Default parameters were used for all software above, except a k-mer size of 111 bp was used. The *N*_50_ and *N*_90_ scaffold sizes are 212,432 bp and 31,095 bp, respectively. The assembled genome has a length of 3,878,578 bp with 159 contigs and an average G+C content of 61.1%.

Genome feature data for *Pseudorhodobacter* sp. strain E13 are presented in Table 1. Genome annotation was performed using the Prokaryotic Genome Annotation Pipeline (10). The draft genome sequence of *Pseudorhodobacter* sp. E13 has 49 RNAs (43 tRNAs, 3 rRNAs, and 3 noncoding RNAs [ncRNAs]), 3,646 protein-coding genes, and 100 pseudogenes. Strain E13 possesses a gene cluster that includes three urease structural proteins (*ureABC*) and four accessory proteins (*ureDEFG*) with a urease transporter (*urtABCDE*). Additionally, genes encoding urea amidolyase for ATP‐dependent cleavage of urea into ammonia and CO_2_ were found (EGN72_15330 and EGN72_15335).

**TABLE 1:** Genome features of *Pseudorhodobacter* sp. strain E13

Genome feature	Value
Genome size (bp)	3,896,924
G+C content (%)	61.85
Total no. of rRNAs (no. of 5S, 16S, 23S rRNAs)	3 (1, 1, 1)
No. of tRNAs	43
No. of noncoding RNAs	3
No. of protein-coding genes	3,722
No. of pseudogenes	48

### Data availability.

This whole-genome shotgun project has been deposited at DDBJ/ENA/GenBank under the accession number NZ_RPEN00000000, BioProject accession number PRJNA505240, and BioSample accession number SAMN10413278. The Sequence Read Archive raw reads are deposited under accession number SRR8599754. The version described in this paper is the first version, NZ_RPEN01000000.
